# Case report: A particularly rare case of endogenous hyperinsulinemic hypoglycemia complicated with pregnancy treated with short-acting somatostatin analog injections

**DOI:** 10.3389/fendo.2022.964481

**Published:** 2022-09-15

**Authors:** Ádám Barsi, Artúr Beke, Beatrix Sármán

**Affiliations:** ^1^ Division of Endocrinology and Metabolism, Department of Internal Medicine and Hematology, Faculty of Medicine, Semmelweis University, Budapest, Hungary; ^2^ Department of Obstetrics and Gynecology, Faculty of Medicine, Semmelweis University, Budapest, Hungary

**Keywords:** endogenous hyperinsulinemic hypoglycemia, nesidioblastosis, pregnancy, octreotide, case report

## Abstract

Hyperinsulinemic hypoglycemia is a rare disease, and only two cases complicated with pregnancy were published previously when our patient became pregnant. We introduce a successful management of a pregnancy in a patient with endogenous hyperinsulinemic hypoglycemia, a condition also known as non-insulinoma pancreatogenous hypoglycemia syndrome or formerly as nesidioblastosis. A 29-year-old female patient was treated with endogenous hyperinsulinemic hypoglycemia since the age of 4 months, taking daily 3 × 75 mg diazoxide, which adds up to 225 mg per day. Adequate glycemic control could be achieved with this therapy. Genetic testing and various imaging examinations were carried out earlier to specify the disease and to exclude focal forms. The patient came to the clinic with a positive pregnancy test and consequential hypoglycemic episodes. Hospital admission was needed to correct the metabolic condition. Although the patient was informed about the potential risks, she decided to carry out the pregnancy. According to the quite limited literature, somatostatin analogs are the only therapy used previously during pregnancy in hyperinsulinemic hypoglycemic patients. One publication reported normal pregnancy outcomes, but in another case, restricted fetal growth was observed. In our case, we stopped diazoxide and parallelly introduced short-acting somatostatin analog octreotide in the therapy, and further dietetic changes were proposed. In addition to daily regular self-blood glucose monitoring, regular gynecological controls were carried out monthly, and healthy fetal development was confirmed. The patient gave birth to her first child, a well-developed female neonate, in the 38th week, by a cesarean section.

## Introduction

Endogenous hyperinsulinemic hypoglycemia is a rare condition characterized by hypoglycemic episodes caused by autonomous insulin secretion without the presence of an insulinoma.

The histological finding is Langerhans’ cell hyperplasia with nesidioblastosis (neo-formation of these cells from the ductal epithelium of the pancreas) (1, 2). The older nomenclature (nesidioblastosis) originates from this histological description (3).

While the congenital form of hyperinsulinemic hypoglycemia is a common cause of persistent hypoglycemia in neonates—termed “persistent hyperinsulinemic hypoglycemia of infancy”—it is uncommon in adolescence or even in adulthood (4). In specific forms, the severity of the disease reduces with age (5).

Focal and diffuse forms of the disease are known, requiring distinct therapeutic approaches. Genetic background is still not fully understood. Specific genetic mutations leading to some of these forms are published. The recessive mutations of the paternal allele of ATP-sensitive K-channel subunit genes (ABCC8 and KCNJ11) have a relatively high specificity (90%) and sensitivity (97%) for focal forms of the disease (6).

As a non-invasive method, ^18^F-DOPA-PET-CT is the best known way to differentiate between focal and diffuse manifestations of endogenous hyperinsulinemic hypoglycemia (7).

The first-line therapy should be *diazoxide*. In some cases, short-acting somatostatin analog *octreotide* was also effective, but the available data are very limited (8, 9).

Focal forms may be treated surgically. In diffuse forms, partial or near-total pancreatectomy could be considered. In mild cases, dietary modifications could be enough to avoid hypoglycemic episodes (10).

The problem arises in women of childbearing age. There are no available data about the effect of diazoxide on human intrauterine development, but some animal research results showed that it passes through the placenta (11). Experimental results in animals suggest that it might cause teratogenic malformations like skeletal and cardiac anomalies and degeneration of pancreatic beta cells (11). When given to neonates and infants, diazoxide is also shown to cause reversible pulmonary hypertension according to some post-marketing reports (11). The United States Food and Drug Administration (U.S. FDA) ranked earlier diazoxide in pregnancy risk category “C”, which means that adverse effects were observed during animal reproduction studies, but there are no adequate human data (11).

We present a case of a pregnancy in a patient with hyperinsulinemic hypoglycemia.

## Case description and diagnostic assessment

The patient had recurrent hypoglycemic episodes since 4 months of age, and diazoxide treatment was introduced (with a daily dose built up to 3 × 3 of 25-mg tablets, which equals 225 mg). Further diagnostic examinations supported the diagnosis of endogenous hyperinsulinemic hypoglycemia.

To exclude focal forms, abdominal ultrasound, CT scan, and a gold standard imaging technique ^18^F-DOPA-PET-CT (7) were performed. None of these showed signs of a focal disease or insulinoma.

Genetic testing gave negative results regarding ABCC8, GCK, HNF1A, HNF1B, INS, and KCNJ11 gene mutations.

Her family history was negative.

Ammonia levels were consequently normal, so hyperinsulinemia/hyperammonemia syndrome could also be excluded (**Table 1**).

**Table 1:** Serum levels of ammonia, glucose, insulin, and C-peptide at various ages and daily dose of diazoxide therapy.

Age	Metabolic parameters (with normal ranges)					Daily diazoxide dose
	Ammonia μmol/L(10–60)	Glucose	Insulin μU/ml (2.6–24.9)	C-peptid eng/ml(0.8–4.2)	Ammonia μmol/L(10–60)	
		mmol/L (3.5–5.5)	mg/dl (63.0–99.0)			
15 years	7.0	3.3	59.4	–	–	200 mg
16 years	7.0	3.6	64.8	–	–	200 mg
17 years	8.0	3.9	70.2	–	–	175 mg
18 years	13.0	2.9	52.2	–	–	200 mg
26 years	–	3.3	59.4	28.46	2.79	225 mg
29 years	33.0	1.5	27	38.73	3.22	50 mg*
Pregnancy	–	5.4	97.2	13.10	2.09	Octreotide**

* Current admission; diazoxide dose was arbitrarily reduced by the patient because of the pregnancy.

** During pregnancy, after 5 months of octreotide therapy (300 μg daily).

During infancy, the patient was not exposed to any provoking factors like drugs and viral infections, which made insulin autoimmune syndrome (IAS) unlikely. Insulin autoantibody measurement at the clinic was not available at the time of diagnosis, and predisposing HLA status was not yet known (12).

At the age of 13 years, the patient went underwent spinal fusion surgery because of scoliosis.

At the age of 23 years, malignant melanoma was surgically removed from her right knee region. Further treatment was not needed.

The patient had her first pregnancy at the age of 26, which had to be terminated on the 13th week predominantly because of life-threatening, uncontrollable maternal hypoglycemic events.

At the age of 29, the patient attended the clinic because of a positive pregnancy test. At the time of clinical admission, the patient was 9 weeks pregnant. When the patient had a positive pregnancy test at home, she decided to lower the dose of diazoxide from 9 tablets (225 mg) daily to only 2 tablets (50 mg). Her blood glucose levels started to get lower, so she compensated with nutritional changes by having a carbohydrate-rich meal every 1–2 h. Hypoglycemic episodes still occurred, so she applied to our clinic for further treatment options.

Basic anthropometric data of our female patient were the following at admission: weight 66 kg, height 163 cm, and body mass index (BMI) 24.8 kg/m^2^.

Blood samples were taken at the time of admission, and as previously found, severe hypoglycemia with measurable insulin level and C-peptide level was present (**Table 2**).

**Table 2:** Pregnant patient’s values of glucose homeostasis at admission.

Pregnant patient’s values at admission		Normal ranges
Glucose	1.5 mmol/L (27 mg/dl)	4.10–5.90 mmol/L (73.8–106.2 mg/dl)
Insulin	38.73 μU/ml (277.89 pmol/L)	2.60–24.90 μU/ml (18.06–172.92 pmol/L)
C-peptide	3.22 ng/ml	0.80–4.20 ng/ml (for euglycemia)

We corrected hypoglycemia with intravenous glucose infusions, and for further clinical observation, we hospitalized the patient until a decision is made about the pregnancy. Blood glucose level was checked every 1–1.5 h by self-check blood glucometer, and if the patient had symptoms or low values, laboratory testing was done. The patient had to eat carbohydrates every 1 or 2 h to avoid hypoglycemia.

β-Human chorionic gonadotropins (β-hCG) measurement supported the pregnancy (166,946 U/L). Gynecological examination, including transvaginal sonography, confirmed the intrauterine pregnancy and established the gestational age.

Genetic consultation was organized to evaluate potential harm to the fetus. The patient was informed about the risks and possible complications of her condition and the available data about octreotide treatment during pregnancy, and she decided to keep the pregnancy.

### Therapeutic intervention

As soon as the patient decided to keep the pregnancy, therapeutic modifications were done since the patient still had hypoglycemic events.

According to the very limited data, somatostatin analogs were used previously during pregnancy in patients with endogenous hyperinsulinemic hypoglycemia without serious side effects (13).

We started subcutaneous somatostatin analog octreotide and stopped diazoxide treatment.

We built up the daily dose of octreotide starting from 3 × 25 μg; then we increased it to 3× 50 μg and then to 3× 100 μg without any side effects. During the titration period, we observed only one severe hypoglycemic episode, a nocturnal event, which needed external intervention (infusion of 10% glucose solution). We measured a serum glucose level of 2.3 mmol/L (41.4 mg/dl).

We consulted with dietitians, and dietary changes were also done. A “high-in-carbohydrate” diet was continued with appropriately adjusted protein and lowered fat intake. The patient was proposed to eat every 1–1.5 h including during nighttime, which meant about 20 occasions per day during the first trimester of pregnancy. Later on, the frequency of food intake could be reduced to every 2–3 h per day, and most of the time, night snacks (11 p.m.–6 a.m.) could be avoided. The three mean mealtimes were preserved (50–70 g of carbohydrates) with appropriate snacks in between. In case of hypoglycemia, the patient needed to take extra sugar or rapidly absorbed carbohydrates in various forms of her liking.

After the appropriate dose of octreotide was reached and nutritional changes were done, no severe hypoglycemic events happened; however, some manageable hypoglycemic episodes still occurred. It led us to refine the dosage to 100–50–50–100 μg accordingly every 6 h. With this regimen, we could reach adequate glycemic control. After the 14th week of pregnancy, we could step back for a 100-μg dose every 8 h, and food intake could be reduced as mentioned above. This therapy did not need more changes in the second and third trimesters.

### Follow-up and outcomes

The patient was released from the clinic after 36 days of admission, and she was called back for check-ups (endocrine and gynecological) monthly.

The patient’s glycated hemoglobin (HbA1c) was 5.2% or 34 mmol/mol (normal range, 20.0–42.0 mmol/mol). With self-blood glucose measurements, only a few hypoglycemic episodes (around 3.00 mmol/L (54 mg/dl)) were detected without clinical symptoms, and these were corrected by additional carbohydrate intake. After the first trimester, the 3 × 100 μg daily octreotide dose remained unchanged until delivery and during the breast-feeding period. With the frequent daily mealtimes, the patient gained a total of 15 kg during pregnancy, and her BMI was 30.5 kg/m^2^ at delivery.

**Figure 1:**
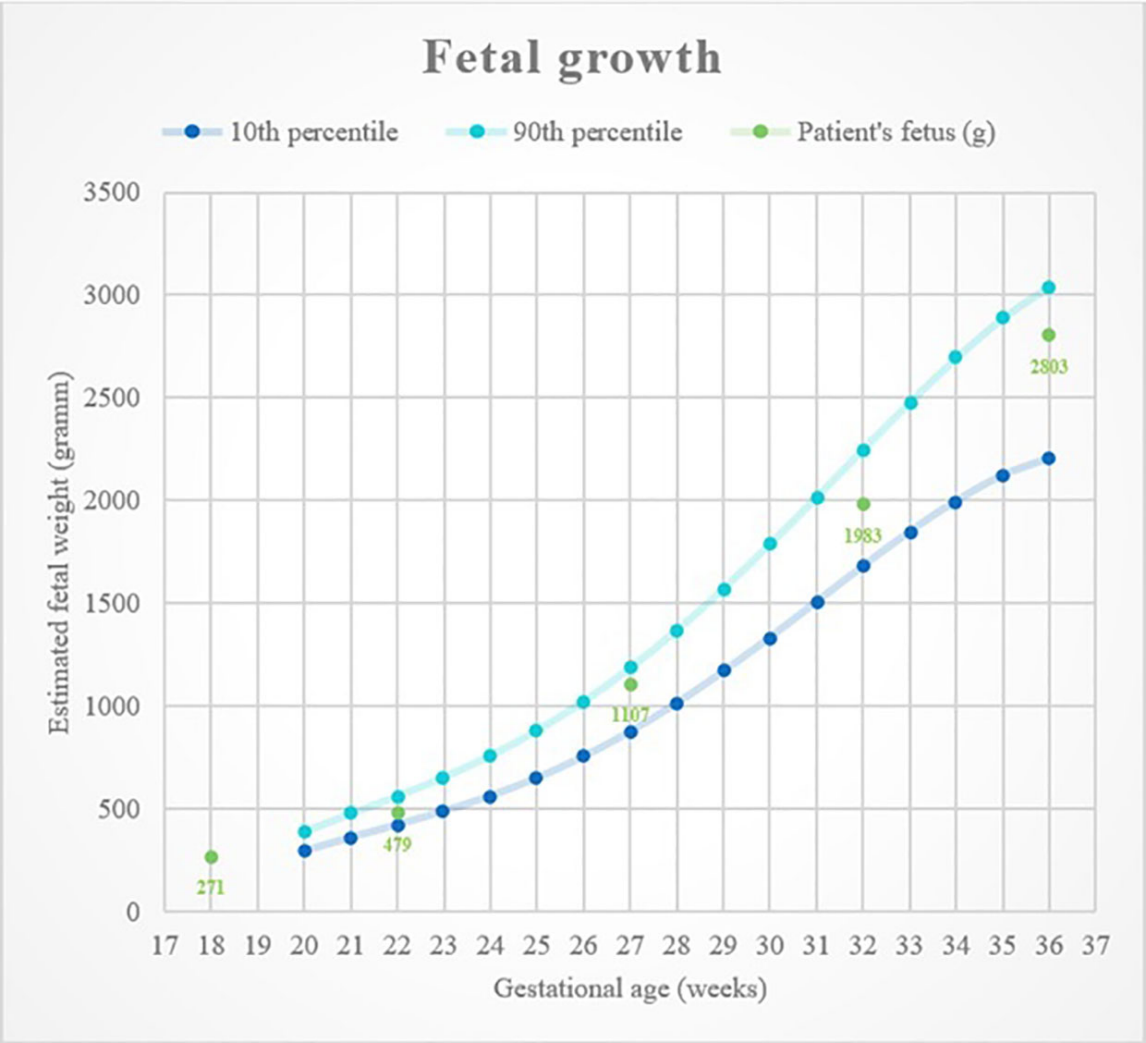
Fetal growth with percentile curves.

Fetal genetic ultrasound was carried out every month, and cardiotocography (CTG) was performed regularly after the 35th week (**Figure 1**). The fetal development was normal.

The mother gave birth to her female neonate on the 38th week of pregnancy by a cesarean section. Indications included transverse position, surgically fixated vertebrae of the mother, and the risk of hypoglycemia. The delivery went without any complications. The infant weighed 2,800 g. Her somatic and mental development is intact (for 7 months now).

The neonatologist recommended breast-feeding, and no contraindications were set for a continuation of the octreotide therapy. The patient remained on somatostatin analog therapy since then, and no severe hypoglycemic episodes were reported.

## Discussion

We reported a normal pregnancy outcome with the short-acting somatostatin analog octreotide in a patient with endogenous hyperinsulinemic hypoglycemia. In our experience, diazoxide therapy was replaceable with somatostatin analog octreotide.

Earlier, these patients were advised against pregnancy because of high hypoglycemic risk and because there were no sufficient data about the ideal management of the condition during pregnancy. Our case shows the possibility for selected patients with endogenous hyperinsulinemic hypoglycemia for childbearing.

The clinical data about somatostatin analog in pregnancy are still very limited in the literature. Animal experiments did not reveal any teratogenic effects of the drug (14). Geilswijk et al. however reported a case where restricted fetal growth was observed in an infant whose mother was treated with octreotide because of hyperinsulinemic hypoglycemia (15). On the contrary, Boulanger et al. described a normal pregnancy in a similar case (13).

There are some other publications about patients with acromegaly treated with somatostatin analogs during pregnancy. Maffei et al. reported an acute decrease in uterine blood supply after octreotide injection, but it did not affect the fetal development or the pregnancy outcome (16). Menis et al. applied *lanreotide* for 1 month and reported an uneventful pregnancy (16).

Long-acting somatostatin analogs may also be considered a therapeutic option (17).

## Patient perspective

The patient is still treated with octreotide instead of the earlier-applied diazoxide.

The mother feels that her mood is significantly better since octreotide therapy even though she has to give herself subcutaneous injections instead of oral medication. Earlier, she experienced depressive periods on a monthly basis while she was taking diazoxide therapy. Since the therapy changed, such episodes did not occur. It is yet unclear whether it is due to the medication or the change in living conditions.

She plans to have a second child, though it is necessary to draw conclusions from this pregnancy and reevaluate the possible risks.

## Conclusion

Our case presents successful management of pregnancy with somatostatin analog octreotide in a patient with endogenous hyperinsulinemic hypoglycemia.

Patients were often advised to avoid pregnancy because of the high risk of hypoglycemia and because adequate protocols are missing. Since it is a very rare condition, and the literature is very limited, only two case reports are available.

There are some data about somatostatin analogs used in patients with acromegaly during pregnancy; however, no therapeutic guideline is available.

Our case provides a possible treatment option for patients living with endogenous hyperinsulinemic hypoglycemia.

## Data availability statement

The raw data supporting the conclusions of this article will be made available by the authors, without undue reservation.

## Ethics statement

Written informed consent was obtained from the individual(s) for the publication of any potentially identifiable images or data included in this article.

## Author contributions

AdB, BS researched data and wrote the manuscript. BS, AdB, ArB contributed to the discussion. ArB contributed to patient examination and obstetrical follow-up. BS reviewed the manuscript. All authors contributed to the article and approved the submitted version.

## Conflict of interest

The authors declare that the research was conducted in the absence of any commercial or financial relationships that could be construed as a potential conflict of interest.

## Publisher’s note

All claims expressed in this article are solely those of the authors and do not necessarily represent those of their affiliated organizations, or those of the publisher, the editors and the reviewers. Any product that may be evaluated in this article, or claim that may be made by its manufacturer, is not guaranteed or endorsed by the publisher.
